# Biosafety and biohazard considerations of HSV-1–based oncolytic viral immunotherapy

**DOI:** 10.3389/fmolb.2023.1178382

**Published:** 2023-09-12

**Authors:** Elizabeth Robilotti, Nathalie C. Zeitouni, Marlana Orloff

**Affiliations:** ^1^ Hospital for Special Surgery, New York, NY, United States; ^2^ University of Arizona College of Medicine and US Dermatology Partners, Phoenix, AZ, United States; ^3^ Thomas Jefferson University Hospital, Philadelphia, PA, United States

**Keywords:** oncolytic immunotherapy, HSV-1, oncolytic virotherapy, biosafety, review

## Abstract

Oncolytic viral immunotherapies are agents which can directly kill tumor cells and activate an immune response. Oncolytic viruses (OVs) range from native/unmodified viruses to genetically modified, attenuated viruses with the capacity to preferentially replicate in and kill tumors, leaving normal tissue unharmed. Talimogene laherparepvec (T-VEC) is the only OV approved for patient use in the United States; however, during the last 20 years, there have been a substantial number of clinical trials using OV immunotherapies across a broad range of cancers. Like T-VEC, many OV immunotherapies in clinical development are based on the herpes simplex virus type 1 (HSV-1), with genetic modifications for tumor selectivity, safety, and immunogenicity. Despite these modifications, HSV-1 OV immunotherapies are often treated with the same biosafety guidelines as the wild-type virus, potentially leading to reduced patient access and logistical hurdles for treatment centers, including community treatment centers and small group or private practices, and healthcare workers. Despite the lack of real-world evidence documenting possible transmission to close contacts, and in the setting of shedding and biodistribution analyses for T-VEC demonstrating limited infectivity and low risk of spread to healthcare workers, barriers to treatment with OV immunotherapies remain. With comprehensive information and educational programs, our hope is that updated biosafety guidance on OV immunotherapies will reduce logistical hurdles to ensure that patients have access to these innovative and potentially life-saving medicines across treatment settings. This work reviews a comprehensive collection of data in conjunction with the opinions of the authors based on their clinical experience to provide the suggested framework and key considerations for implementing biosafety protocols for OV immunotherapies, namely T-VEC, the only approved agent to date.

## Introduction

Oncolytic viruses (OVs) are a form of oncolytic immunotherapy (OI) for cancer that utilizes native or genetically modified viruses that preferentially replicate in and kill tumors and initiate host antitumor immunity (Kaufman and Bommareddy, 2019). During the last 20 years, there have been a substantial number of clinical trials evaluating OV immunotherapies across a broad range of cancers (Macedo et al., 2020; Salloum et al., 2021).

Four OVs have received regulatory approval to date in various global markets. Rigvir (ECHO-7) was the first to gain regulatory approval (Latvia, 2004) and is a member of the enteric cytopathogenic human orphan type 7 picornavirus virus group. Rigvir did not undergo genetic modification but rather was selected and adapted for the treatment of melanoma (Alberts et al., 2018). Oncorine (H101), the first recombinant OV to gain regulatory approval (China, 2005), is an attenuated serotype 5 adenoviral vector with viral E1B-55k deleted and 4 deletions in viral E3, designed to treat head and neck cancer in combination with chemotherapy (Russell and Peng, 2018). Teserpaturev/G47Δ is a triple-mutated recombinant herpes simplex virus type 1 (HSV-1) that received conditional, time-limited approval in Japan in 2021 for the treatment of glioma (Frampton, 2022). Finally, talimogene laherparepvec (T-VEC) is the only OV immunotherapy approved by the US Food and Drug Administration (FDA; 2015) or by the European Medicines Agency (2015) and is used to treat patients with unresectable advanced melanoma recurrent after initial surgery (European Medicines Agency, 2015; Amgen Inc., 2017). T-VEC has also been approved for treatment of melanoma in Australia and Israel (Shalhout et al., 2023). T-VEC is a category 1 recommendation in the National Comprehensive Cancer Network guidelines for the treatment of stage III cutaneous melanoma (National Comprehensive Cancer Network, 2022). T-VEC is an oncolytic HSV-1 modified through viral gene deletions and expression of human granulocyte-macrophage colony-stimulating factor (GM-CSF) (Andtbacka et al., 2015). Many OV immunotherapies in clinical development are similarly based on HSV-1 (Thomas et al., 2019; Macedo et al., 2020).

While HSV-1 serves as the backbone for many OV immunotherapies, approximately 50%–80% of adults (≥30 years of age) in America have preexisting antibodies to HSV-1, which is most commonly responsible for oral herpes that causes sores or blisters in or around the mouth (McQuillan, 2018; Ayoub et al., 2019; Johns Hopkins Medicine, 2022). HSV-1 infection can also cause herpetic whitlow (a painful infection of the finger), genital ulcers, encephalitis, and corneal blindness (Brady and Bernstein, 2004). HSV-1 is not an airborne virus, and transmission requires direct contact with the lesion or mucosal secretions from the infected individual (Brady and Bernstein, 2004; Fatahzadeh and Schwartz, 2007). HSV-1 infection can respond well to antiviral therapies such as acyclovir, valaciclovir, and famciclovir (Kimberlin and Whitley, 2007).

For healthcare providers, hospital and medical office personnel, and patients, distinguishing between the OV (eg, T-VEC) and the unmodified wild-type virus (eg, HSV-1) is critical, as the handling and infectivity of genetically modified OV immunotherapies are different than the handling and infectivity of wild-type viruses that cause human infection. As more patients have been treated with OV immunotherapies during the last few years, our understanding of issues surrounding the safety and handling of OV immunotherapies has evolved, and existing biosafety and handling guidelines for OV immunotherapies should also evolve. With an increase in usage of these therapeutics, it is important to review the associated biosafety and biohazard guidelines. In this manuscript, we discuss important characteristics of OV immunotherapies that should be considered during biohazard classification that may help inform guidance on handling these therapeutics. We recommend these designations be determined based on the individual properties and available biosafety evidence for each OV immunotherapy, rather than a blanket assignment imposing the strictest handling requirements based on generalized perceptions of biosafety for handling viruses. As T-VEC is the only approved OV immunotherapy agent to date, much of the guidance included herein may be more closely adapted from T-VEC regulations. Ultimately, it is up to the national biosafety committees to implement appropriate measures for each product in a clinical setting. We focus on key considerations and challenges, including changing existing perceptions regarding safety of viral therapy and detailing logistical hurdles that could reduce patient access to these therapeutics.

### Biosafety levels

National biosafety guidelines (biosafety levels [BSLs]) implemented by the US Centers for Disease Control are assigned to an agent based on infectivity, severity of disease, transmissibility, and the nature of the work being conducted; BSL assignments range from level 1 (most basic level of protection) to 4 (the most restrictive) (Center for Disease Control and Prevention, 2020). The BSL designation describes the microbiological practices, safety equipment, and facility safeguards for the level of risk associated with handling the agent (Table 1) (Center for Disease Control and Prevention, 2020).

**TABLE 1:** Laboratory biosafety levels.

BSL	Agent characteristics	Special practices^a^	Primary barrier and PPE^a^	Facilities^a^
**1^b^**	• Well-characterized	• Standard microbiological practices	• No primary barriers required	• Laboratory doors
	• Not known to consistently cause disease in immunocompetent adults		• Protective laboratory clothing, face, and eyewear, as needed	• Sink for handwashing
	• Minimal potential hazard to laboratory personnel and the environment			• Laboratory bench
				• Windows with screens
				• Lighting adequate for all activities
**2^c^**	• Associated with human disease	• Limited access	• BSCs or other primary containment device used for manipulations of agents that may cause splashes or aerosols	• Self-closing doors
	• Pose moderate hazards to personnel and the environment	• Occupational medical services, including medical evaluation, surveillance, and treatment, as appropriate	• Protective laboratory clothing	• Sink located near exit
		• All procedures that may generate an aerosol or splash conducted in a BSC	• Other PPE, including respiratory protection, as needed	• Windows sealed or fitted with screens
		• Decontamination process for laboratory equipment		• Autoclave available
**3**	• Indigenous or exotic agents	• Access limited to those with need to enter	• BSCs for all procedures with viable agents	• Physical separation from access corridors
	• May cause serious or potentially lethal disease through the inhalation route of exposure	• Viable material removed from laboratory in primary and secondary containers	• Solid front gowns, scrubs, or coveralls	• Access through 2 consecutive self-closing doors
		• Opened only in BSL-3 or animal BSL-3 laboratories	• 2 pairs of gloves, when appropriate	• Hands-free sink near exit
		• All procedures with infectious materials performed in a BSC	• Protective eyewear and respiratory protection, as needed	• Windows are sealed
				• Ducted air ventilation system with negative airflow into laboratory
				• Autoclave available, preferably in laboratory
**4**	• Dangerous and exotic agents that pose high individual risk of aerosol-transmitted laboratory infections and life-threatening disease	• Clothing change before entry	• BSCs for all procedures with viable agents	• Entry sequence
	• Infections are frequently fatal, for which there are no vaccines or treatments	• Daily inspections of essential containment and life support systems	• Solid front gowns, scrubs, or coveralls	• Entry through airlock with airtight doors
	• Related agents with unknown risk of transmission	• All wastes decontaminated prior to removal from laboratory	• Gloves and/or full-body, air-supplied, positive-pressure suit	• Walls, floors, ceilings form sealed internal shell
		• Shower on exit		• Dedicated, non-recirculating ventilation system required
				• Double-door, pass-through autoclave required

Adapted from the Biosafety in Microbiological and Biomedical Laboratories 6th Edition (Center for Disease Control and Prevention, 2020).

^a^ Each successive BSL contains the recommendations of the preceding level(s).

^b^ Recommended BSL for handling small volumes (<10 L) of T-VEC (Amgen Inc., 2018; Andtbacka et al., 2019a).

^c^ Recommended BSL for handling large volumes (>10 L) of T-VEC (Amgen Inc., 2018).

BSC, biological safety cabinet; BSL, biosafety level; PPE, personal protective equipment; T-VEC, talimogene laherparepvec.

The current biosafety guidelines suggest that BSL-2 facilities with additional containment and safety procedures, such as those described for BSL-3, should be considered when producing, purifying, and concentrating wild-type HSV-1 (Center for Disease Control and Prevention, 2020). However, the current guidelines also recognize that human herpes viruses have not demonstrated a high potential hazard for laboratory-associated infections (Center for Disease Control and Prevention, 2020). As these BSL guidelines are specific to wild-type HSV-1, they may not be appropriate for the attenuated, genetically modified OV immunotherapies utilizing the HSV-1 backbone for cancer therapy. The BSL guidelines serve as recommendations to each institution’s biosafety, infection control/prevention, and occupational health committees that, in turn, provide guidance to staff on how to handle the respective agent (in this case, the OV immunotherapy). Additionally, OV immunotherapies are often treated as hazardous drugs, and handling guidelines may adhere to the US Pharmacopeia (USP) guidelines for handling hazardous drugs in healthcare settings (USP800) (Soefji, 2013; The United States Pharmacopeial Convention, 2020). The USP800 contains strict handling guidelines, such as preparation of hazardous drugs in a designated ventilated hood that is in a space separated from other preparations and the use of double chemotherapy gloves (The United States Pharmacopeial Convention, 2020).

The material safety data sheet issued by the manufacturer of T-VEC recommends different BSL containments based on the volume handled; this separates actions dealing with large volumes, such as manufacturing or mixing, from handling small volumes, such as the administration of single-dose tumor-directed injections for patients (Amgen Inc., 2018). Accordingly, BSL-2 containment procedures are recommended for tasks performed with volumes >10 L, and BSL-1 containment and work practices are recommended for research activities handling small volumes (Amgen Inc., 2018). As such, it may be permissible to handle T-VEC at the injection site following BSL-1 practices with universal precautions, such as appropriate hand hygiene and the use of personal protective equipment (PPE), practiced as recommended for single-dose T-VEC administration (which is packaged in 1 mL vials and administered at a volume of up to 4 mL) (Amgen Inc., 2018). Healthcare workers who are immunocompromised or pregnant should not prepare or administer T-VEC (Amgen Inc., 2017). Despite this seeming differentiation between preparation of large volumes and the single-use injection, blanket BSL-2 containment guidelines are often implemented for handling all OV immunotherapies, including T-VEC, frequently leading to logistical hurdles at treatment centers. Prior to an agent’s FDA approval, an internal biosafety committee may provide the same guidance to both the pharmacy and clinical staff. For example, the Oncology Nursing Society guidelines for handling OV immunotherapies indicate that OV immunotherapy–only clinic days or patient rooms should be considered, thus reducing the availability of facilities and staff, even though there is no evidence of risk or spread to healthcare workers or other patients during or following administration of T-VEC to patients (Oncology Nursing Society, 2022). Increasing the awareness of the differences between wild-type viruses and attenuated, genetically modified OV immunotherapies may serve as the key to altering misconceptions surrounding the BSL containment guidelines for virus-based OIs. Further, emerging data from biosafety analyses of OV immunotherapies under clinical evaluation will be of importance for establishing specific BSL requirements for individual agents.

### Naturally occurring and genetically modified OVs

**FIGURE 1:**
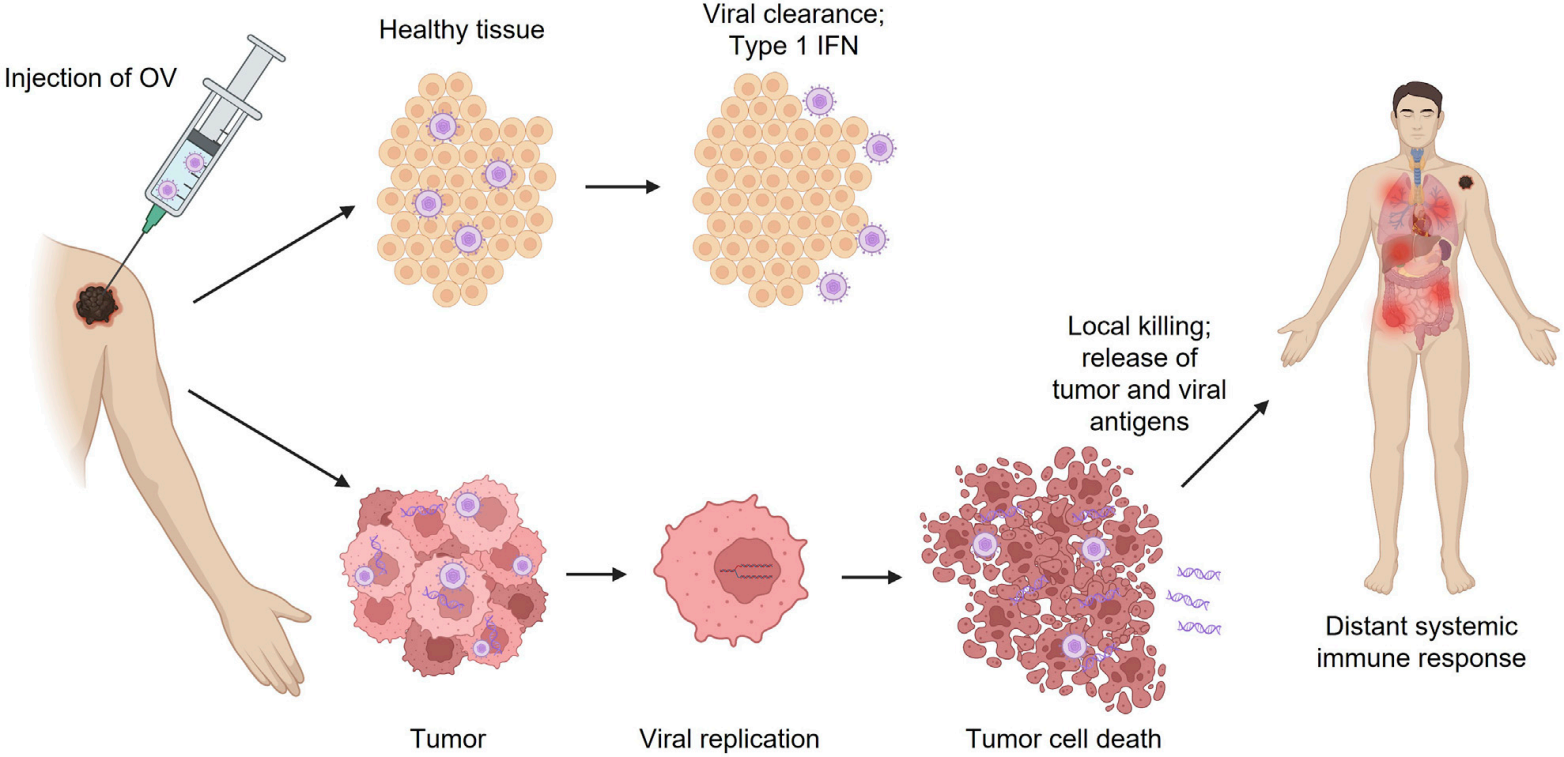
Effects of OV immunotherapy injection in tumor vs. healthy tissue. IFN, interferon; OV, oncolytic virus.

Wild-type, unmodified viruses may be selected for development into OVs based on their natural propensity for antitumor activity or oncotropism. These viruses may target specific molecular mechanisms that are either altered or overexpressed in tumors to preferentially infect and kill cancer cells without affecting surrounding healthy cells (Wollmann et al., 2012). Normal, healthy cells utilize a variety of intracellular pathways to detect and eliminate viruses; these pathways are often abnormal in tumors, which leads to their natural vulnerability to viral infection (Kaufman et al., 2015; Maroun et al., 2017). Specifically, in response to viral infection, healthy cells can activate the interferon-induced, double-stranded RNA-activated protein kinase R (PKR) pathway, which inhibits protein translation and prevents the production of viral progeny, thus stopping the spread of the virus (Figure 1) (Meurs et al., 1990; Kaur et al., 2012; Kaufman et al., 2015). This pathway is not initiated in many cancers due to impaired interferon signaling, making those tumors vulnerable to viral lytic replication, spread, and destruction (Figure 1) (Critchley-Thorne et al., 2009; Kaufman et al., 2015). Reovirus is an example of a naturally occurring OV assessed in clinical studies for the treatment of various cancers, including advanced solid tumors and brain tumors (Forsyth et al., 2008; Harrington et al., 2010b; Lolkema et al., 2011; Mondal et al., 2020). Other examples of naturally occurring OVs include the Newcastle disease virus, parvovirus H-1, Alpha virus M1, and picornavirus-based viruses (Mondal et al., 2020). The mechanism of action of OV immunotherapy has been extensively reviewed in previous publications (Kaufman et al., 2015; Bommareddy et al., 2018).

When OVs are genetically modified, many of the harmful characteristics of the original virus are lost, and the result is a therapeutic product that could potentially be similar to live-attenuated vaccines for infectious diseases. Using genetic engineering techniques, wild-type viruses can be attenuated by deleting genes associated with pathogenicity in normal healthy tissue; this can aid in making OVs more selective for tumors and reduce off-target effects (eg, replication in normal healthy tissue; Figure 1) (Jhawar et al., 2017). The HSV-1 genome is large (∼150 kilobases) and encodes many genes that are not essential for replication; this allows researchers to manipulate the HSV-1 genome without hindering the ability to replicate in tumors (Peters and Rabkin, 2015). Therefore, genetically modified HSV-1 OV immunotherapies, such as T-VEC, G47Δ, HSV1716, G207, and NV1020, which all have alterations in the infected cell protein (ICP) 34.5 so-called “neurovirulence factor”–encoding genes that prevent effective replication in normal tissue, have been widely studied in clinical trials for the treatment of various types of malignancies (Geevarghese et al., 2010; Markert et al., 2014; Andtbacka et al., 2015; Streby et al., 2017; Andtbacka et al., 2019b; Todo, 2019).

The most commonly used HSV-1–based OV immunotherapy, FDA-approved T-VEC, has deletions in the genes encoding ICP34.5 and ICP47 (Hu et al., 2006; Andtbacka et al., 2015). ICP34.5 is a neurovirulence factor that plays a critical role in viral replication, allowing HSV-1 to overcome the host cell’s ability to shut down protein synthesis in response to viral infection via the PKR pathway (He et al., 1997; Aldrak et al., 2021). Because interferon signaling and the activation of PKR are often impaired in tumors, deletion of ICP34.5 serves to spare healthy tissue and increase tumor selectivity (Strong et al., 1998; Liu et al., 2003; Critchley-Thorne et al., 2009; Jhawar et al., 2017; Aldrak et al., 2021). ICP47 helps HSV-1 evade the host immune response by reducing antigen presentation and attenuating CD8^+^ T cell recognition of infected cells (Berger et al., 2000). Deletion of ICP47 restores antigen presentation and increases expression of the US11 gene, which promotes replication of ICP34.5-deleted HSV-1 in tumors without restoring replication in normal tissue (Mohr et al., 2001; Liu et al., 2003). T-VEC is also armed with the gene for human GM-CSF to enhance the systemic antitumor response (Liu et al., 2003; Ferrucci et al., 2021). With these modifications, T-VEC is estimated to be 1:100- to 1:10,000-fold less pathogenic and neurovirulent than wild-type HSV-1 (European Medicines Agency, 2015). Another modified HSV-1 in phase 1 clinical development is rQNestin34.5, which retains expression of the ICP34.5 gene under the transcriptional control of the nestin promoter, thereby enhancing its expression selectively in nestin-overexpressing tumors (Chiocca et al., 2020). A newer HSV-1 OV immunotherapy platform utilizes additional modifications to enhance tumor cell killing and amplify the immune response, such as insertion of a gene encoding the fusogenic gibbon ape leukemia virus surface glycoprotein with the R sequence deleted and expression of an anti–cytotoxic T-lymphocyte antigen-4 antibody-like molecule or immune pathway–activating ligands (Thomas et al., 2019). Other potential targets to modify HSV-1 for specificity and safety that have been explored in other platforms in clinical trials include ICP6, thymidine kinase, uracil DNA glycosylase, US3, and UL56 (Peters and Rabkin, 2015).

A potential concern for genetically modified OV immunotherapies is the reversion to wild-type virus. However, there is no definitive evidence of reversion to wild-type virus in treated patients to date (Kaufman et al., 2015; Maroun et al., 2017). Notably, OV immunotherapies can be developed from known human pathogens for which effective antiviral therapies exist (such as HSV).

### Safety, biological shedding, and close contact transmission

With the aforementioned modifications to HSV-1, it is important to show that HSV-1–based OV immunotherapies are well tolerated clinically and do not pose a risk to healthcare workers and close contacts (eg, caregivers, family members). OV immunotherapies in general have been associated with a tolerable safety profile with common treatment-related adverse events (AEs), including low-grade systemic symptoms (fever, chills, flu-like symptoms, etc.) and local injection-site reactions (Macedo et al., 2020). This favorable safety profile may be attributed to the fact that OV immunotherapies are often delivered via localized intratumoral injection, unlike systemic immunotherapies, which are associated with higher incidences and severities of AEs (De Lombaerde et al., 2021). In the primary analysis of a phase 3 study that evaluated T-VEC in patients with unresectable stage IIIB–IV melanoma, the most common (>25% of patients receiving T-VEC) AEs were fatigue, chills, pyrexia, nausea, flu-like illness, and injection-site pain; the only grade 3/4 AE that occurred in >2% of patients receiving T-VEC was cellulitis (2.1%), and there were no fatal treatment-related AEs (Andtbacka et al., 2015). In the T-VEC arm, 16 patients (5.5%) developed an HSV-1 infection compared with 2 patients (1.6%) in the GM-CSF arm (European Medicines Agency, 2015). Patients receiving T-VEC had a significantly higher durable response rate (primary endpoint) compared with patients receiving control GM-CSF injections (16.3% vs. 2.1%) (Andtbacka et al., 2015). Similarly, another HSV-1–based agent in clinical development, RP1, was well tolerated and resulted in durable responses in combination with systemic immunotherapy (the programmed cell death protein 1 monoclonal antibody nivolumab) in patients with melanoma and non-melanoma skin cancers (Milhem et al., 2022).

One of the concerns and common misconceptions surrounding the use of OV immunotherapies is the risk of uncontrolled replication and possible transmission to close contacts (Kaufman et al., 2015; Maroun et al., 2017). Therefore, it is imperative that researchers and sponsors make results from shedding/transmissibility analyses conducted during clinical product development readily available to the public, specifically to centers using the product to treat patients, as well as close contacts and caregivers (Center for Biologics Evaluation and Research, 2015). As shedding analyses are uniformly required for clinical OV immunotherapy development, we expect these data to be more readily available to help guide future biosafety considerations for individual agents, particularly for those in advanced stages of development and seeking regulatory approval. Available reports to date have shown little evidence of shedding/transmissibility with HSV-based OV immunotherapies (Mace et al., 2008; Andtbacka et al., 2019a; Todo et al., 2022). A detailed biodistribution, shedding, and transmissibility analysis was conducted for T-VEC using phase 2 data from 60 patients with stage IIIB–IVM1c melanoma (Andtbacka et al., 2019a). While on treatment, T-VEC DNA was detected via quantitative polymerase chain reaction (qPCR) on swabs from the injected lesions (60/60, 100%), in blood (59/60, 98.3%), on swabs from the exterior of occlusive dressings (48/60, 80.0%), in urine (19/60, 31.7%), on swabs from the oral mucosa (5/60, 8.3%), and on swabs from the anogenital area (2/25, 8.0%) (Andtbacka et al., 2019a). T-VEC DNA was detected on swabs only from the surface of injected lesions during the safety follow-up period (14% of patients, 30–60 days after the last T-VEC injection) (Andtbacka et al., 2019a). It is important to note that qPCR can detect extremely low levels of viral DNA; in this particular study, the test was considered positive if DNA was detectable above the assay cutoff values (lower limit of quantification: 1 copy of T-VEC DNA in 76 µg total DNA for blood, 24 µg total DNA for urine, and 18 µg total DNA for swabs), even if the level was too low to be quantified (Andtbacka et al., 2019a).

Most importantly, while DNA was present in the aforementioned swabbed areas, the assay for infectivity (assessment of median tissue culture infectious dose [TCID_50_]) found that potentially infectious virus (T-VEC) was only present in 1.1% (8/740) of samples from the surface of injected lesions, all during cycle 1 or 2, indicating that the live virus does not survive for long outside of host cells (Andtbacka et al., 2019a). Approximately one-third of patients (19/60, 31.7%) developed lesions of suspected herpetic origin; however, only 3 of these patients had detectable T-VEC DNA and none were positive for infectivity (Andtbacka et al., 2019a). Of note, 67% of the global population are estimated to have been infected with HSV-1 by the age of 50 (World Health Organization, 2022). Similar considerations have recently been applied to the presence of SARS-CoV-2 RNA vs. the presence of infectious virus on surfaces; it was concluded that the presence of viral RNA/DNA is not a valid surrogate for the presence of infectious virus, and these results should always be accompanied by assays for infectivity (such as TCID_50_) (Goldman, 2021).

In the above study, 1 investigator was exposed to T-VEC through unprotected skin and did not develop any symptoms. One investigator and 3 close contacts reported signs and symptoms of suspected herpetic infection; however, none had any detectable T-VEC DNA (1 close contact declined testing) (Andtbacka et al., 2019a). Despite concern over the risk of transmission from contact with dressings or treated lesions, this study concluded that there is minimal risk of T-VEC transmission from treated patients to their close contacts and healthcare staff with proper administration and handling (Andtbacka et al., 2019a). Early studies with T-VEC demonstrated similar results; infectious virus was only detectable on the surface of the injected lesions/the inside surface of occlusive dressings, suggesting very low risk of transmission to close contacts when occlusive dressings are applied correctly (Hu et al., 2006; Harrington et al., 2010a; Andtbacka et al., 2019a).

Outside of host cells, HSV-1–based T-VEC is rapidly inactivated and carries little risk of aerosolization in a dermatologic setting; therefore, the potential for exposure from the environment at the site of administration is negligible (European Medicines Agency, 2015). The most likely mechanism of exposure in healthcare staff is thought to be from needlestick injuries during administration (European Medicines Agency, 2015). Needlesticks or exposure to broken skin require thorough cleaning with soap and water or a skin disinfectant; antiviral drugs may be administered prophylactically (Amgen Inc., 2018). A 2015 briefing from the FDA reported only 1 accidental needlestick exposure during the phase 3 T-VEC clinical trial; the healthcare worker developed a herpetic lesion at the needlestick site that resolved following treatment with acyclovir (The U.S. Food and Drug Administration, 2015). In a self-reported survey from healthcare workers, 5 incidences of accidental exposure to T-VEC by needlestick injury or splash were reported across 4,100 treatment visits (Robilotti et al., 2019). Out of these incidents, 1 healthcare worker developed herpetic whitlow following an accidental needlestick that resolved after acyclovir treatment (Robilotti et al., 2019). A postmarketing trial is ongoing to assess the long-term risk of infection and transmission in patients, caregivers, and healthcare workers treated with or exposed to T-VEC (Robilotti et al., 2019; The U.S. National Library Of Medicine, 2022).

In a review of 97 clinical studies using OV immunotherapies, viral shedding analysis was conducted in 71 studies (73.2%), with 26 studies (26.8%) not reporting shedding data (Macedo et al., 2020). The most common sites evaluated for biodistribution were blood or serum, and shedding was analyzed from urine and tumor sites (Macedo et al., 2020). All 71 studies reported evidence of virus detection; most studies (58 [81.7%]) utilized PCR to detect specific viral genome sequences (Macedo et al., 2020). However, only 13 of these 71 studies (18.3%) evaluated the presence of infectious viral particles, indicating a need for more complete shedding and biodistribution analyses among OV immunotherapy clinical trials (Macedo et al., 2020). Of note, no instances of viral transmission to household contacts or healthcare providers were reported among any of the studies.

**FIGURE 2:**
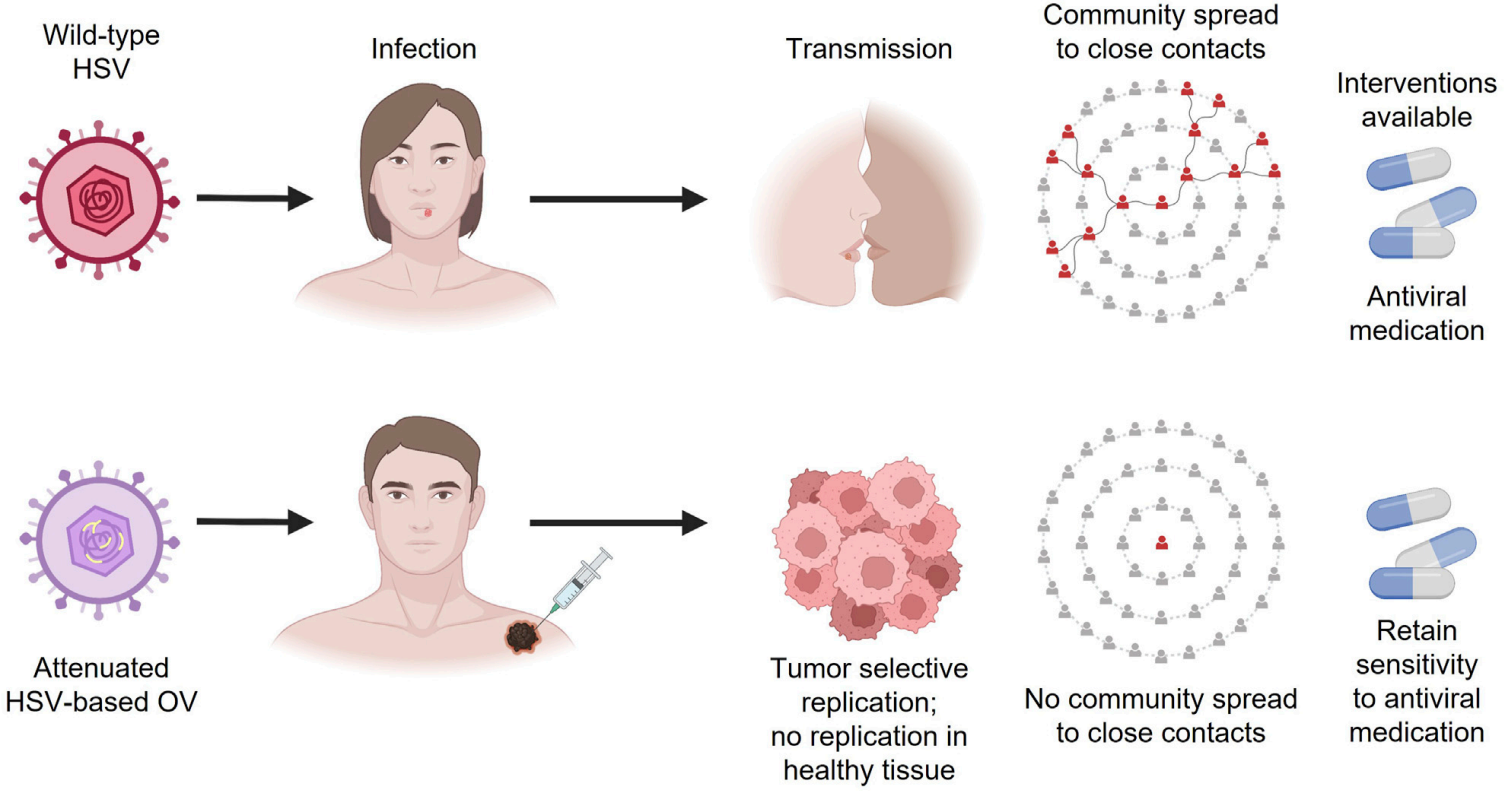
Differences between wild-type HSV and HSV-based OV immunotherapies. HSV, herpes simplex virus; OV, oncolytic virus.

While there is a common misconception that OV immunotherapies may cause infections in healthcare workers and close contacts, the shedding data provided for T-VEC support that this risk is very low, and there is no community spread of HSV-1 due to OV immunotherapy injection (Figure 2). However, bioavailability and shedding studies need to be made readily available to the public. It is worth noting that while T-VEC and many other agents in advanced clinical development are administered intratumorally, several OV immunotherapies in development are designed for intravenous administration (Li et al., 2020). While efficacy data are limited in the case of systemic administration, neutralizing antibodies against the virus, T-cell–mediated antiviral immunity, and the inability of the virus to replicate in blood appear to make these agents relatively safe (Bommareddy et al., 2018; Macedo et al., 2020).

### Biosafety recommendations

**FIGURE 3:**
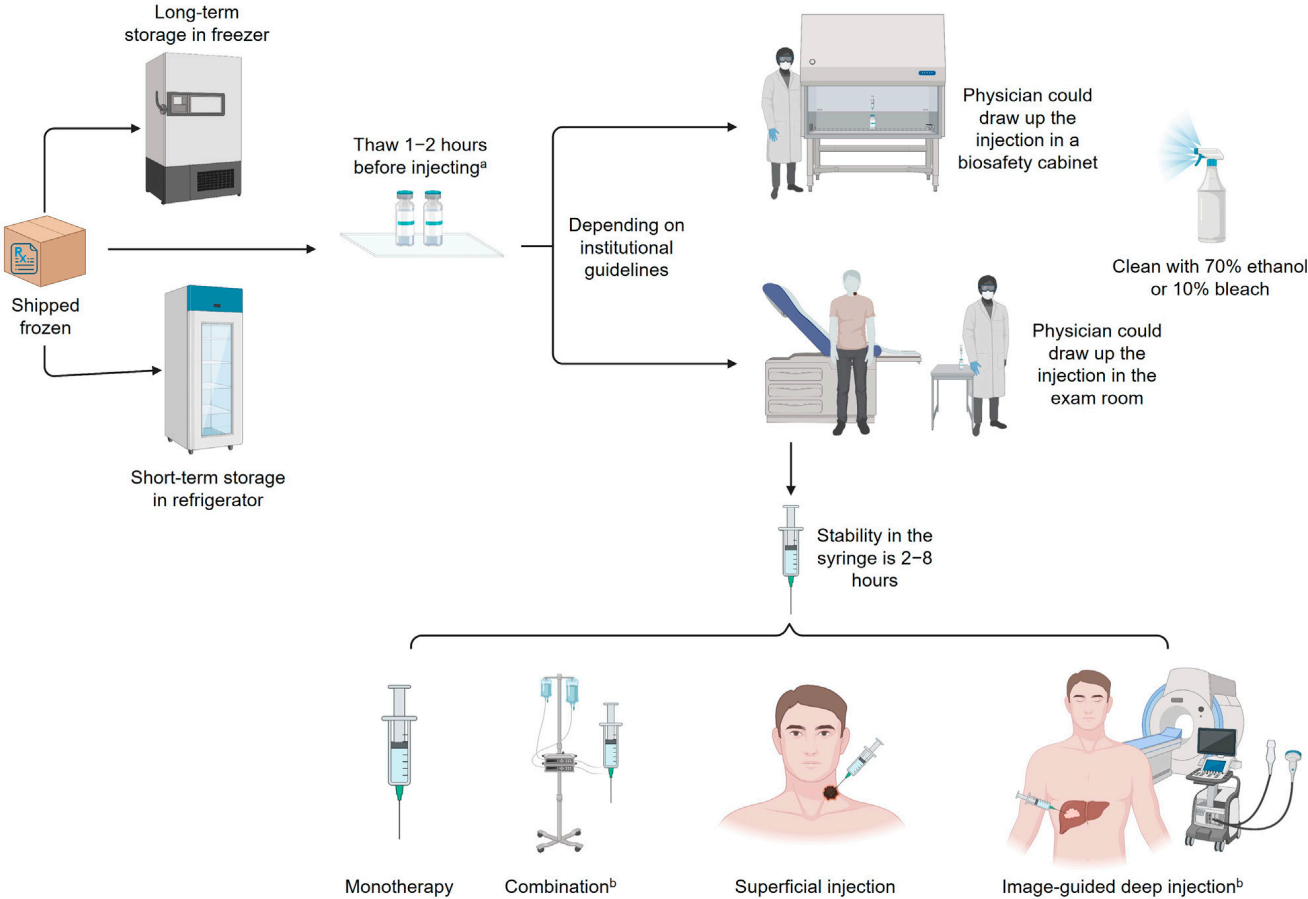
Handling HSV-1 OV immunotherapies. ^a^Stability of each strain varies; 8 h at room temperature is optimal. ^b^Not currently approved for T-VEC; investigational use only. HSV-1, herpes simplex virus type 1; OV, oncolytic virus; T-VEC, talimogene laherparepvec.

The current guidelines for handling intratumoral OV immunotherapies (in this case, T-VEC) in the US are largely based on or influenced by prior clinical trial experience. However, enhanced knowledge about the safety of OV immunotherapies and increased clinical use may warrant updates to current practices in order to reduce barriers to effective cancer treatments partly by alleviating logistical hurdles experienced by treatment centers and healthcare providers (Orloff, 2016). Per the package insert, T-VEC must be stored at −90°C to −70°C, thawed for 30–70 min, and administered within prespecified times dependent on concentration and refrigeration; narrow preparation windows require efficient organization to ensure appropriate scheduling (Figure 3) (Orloff, 2016; Amgen Inc., 2017). Hospital pharmacy workers are often instructed to treat OV immunotherapies like hazardous or investigational drugs, and a separate area/biosafety cabinet is recommended (Soefji, 2013). Chemotherapy agents are classified as hazardous drugs, and contact exposure with these agents causes serious acute side effects such as rash and vomiting; these agents have also been linked to cancer development and reproductive toxicity in exposed healthcare workers (Table 2) (National Institute for Occupational Safety and Health, 2004). Having this designated biosafety cabinet would allow for more streamlined preparation of OV immunotherapies by eliminating extensive disinfection between different types of therapeutics, such as chemotherapy agents (Soefji, 2013). However, additional biosafety cabinets are expensive, require extensive maintenance, and may not be feasible for small pharmacies, outpatient surgery centers, or community practices; furthermore, the available evidence does not support the need for such special precautions with use of T-VEC, and the use of a hood to load syringes may not be necessary. In these settings, depending on institutional guidelines, providers may choose to draw up the injection in the patient room (Figure 3). Contact surfaces can be effectively disinfected with common laboratory cleaning solutions (Figure 3).

**TABLE 2:** Biosafety comparisons between chemotherapy agents and HSV-1–based OV immunotherapies for occupational handling and exposure.

	Chemotherapy agents^a^	HSV-1 OV immunotherapies^b^
Potential exposure health effects	• Rash^c^	• Herpetic lesion/infection
	• Vomiting^c^	• Herpetic whitlow
	• Sore throat^c^	
	• Chronic cough^c^	
	• Dizziness^c^	
	• Headache^c^	
	• Eye irritation^c^	
	• Hair loss^c^	
	• Allergic reactions^c^	
	• Leukemia and other cancers^d^	
	• Reproductive toxicity including infertility, miscarriage, birth defect, and other adverse pregnancy health outcomes^d^	
Capacity for viral replication/transmission	• Not applicable	• Potential capacity for replication/transmission

^a^ Occupational exposure health risks from chemotherapeutic agents (Boiano et al., 2014).

^b^ Occupational exposure health risks from OV immunotherapies (Amgen Inc., 2017; Harrington et al., 2017; Amgen Inc., 2018).

^c^ Potential acute exposure health effects.

^d^ Potential long-term or chronic exposure health effects.

HSV-1, herpes simplex virus type 1; OV, oncolytic virus.

When handling larger volumes (>10 L) of T-VEC during manufacturing, BSL-2 precautions are recommended (Amgen Inc., 2018). However, handling small volumes, such as those used during the actual administration of T-VEC, only requires BSL-1 precautions per manufacturer recommendations; despite this recommendation, guidelines that include BSL-2–level PPE and contact isolation procedures (OV immunotherapy–only patient rooms or treatment bays) are common (Seery, 2017; Amgen Inc., 2018; Oncology Nursing Society, 2022), despite no evidence of such a need. In stark contrast, such procedures are not in place for attenuated live virus vaccines, such as the vaccines for mumps-measles-rubella and influenza (intranasal), which are drawn up and administered in outpatient settings by healthcare workers using standard precautions. Unlike the prescribing information for T-VEC, the information for those vaccines contains no specific occupational hazard or protection guidance for healthcare workers handling those products, despite evidence of viral shedding up to 28 days after vaccination in patients who received the intranasal flu vaccine (Amgen Inc., 2017; Merck Sharp and Dohme Corp, 2020; MedImmune LLC, 2022). The detailed shedding analysis of T-VEC further supports implementation of BSL-1 safety precautions, indicating that it may be more appropriate to consider the shedding profile of an OV immunotherapy and update the BSL classification accordingly (Andtbacka et al., 2019a). We understand, however, that this consideration is contingent upon this information being readily available. With comprehensive information and educational programs, our hope is that the biosafety guidance on OV immunotherapies will evolve, thus alleviating logistical hurdles faced by treatment centers and increasing access to these therapies that have demonstrated promising clinical benefit.

Given the aforementioned data on the low risk of clinically significant transmission, we recommend that BSL-1–level handling procedures, including the appropriate PPE and precautions for immunocompromised individuals, be considered. In that regard, strict contact isolation and handling procedures are not necessary, and these injections can be administered safely in hospitals, as well as in outpatient clinics and medical office settings. However, it will be necessary to consider the individual specification of a given OV immunotherapy, including the type of virus, modifications, mode of administration, and availability of antiviral medications in the final determination of proper handling and administration procedures. It will be essential for sponsors to provide shedding and biodistribution data from clinical studies of OV immunotherapies to help shape these guidelines.

Addressing T-VEC and similar agents as “viruses” or “oncolytic virus therapy” may reinforce the difficulty in separating the therapy from the infection caused by the wild-type virus. For this reason, the authors propose to use the term “oncolytic immunotherapy” or “OI” to refer to oncolytic viral immunotherapy when appropriate, not to conceal the viral basis of these therapies, but rather to highlight the immunologic mechanism of action and mitigate potential nomenclature-based misconceptions and concerns over the risk of transmissibility and infectivity, which has so far been shown to be negligible. The terminology should not be used indiscriminately or to conceal the nature of virus-based therapies. Therefore, while transparency of the viral nature of these therapies remains important, we propose that “oncolytic immunotherapy” or “OI” may be the preferable term to enhance uptake of T-VEC and agents with similar mechanisms of action when communicating with healthcare professionals, caregivers, clinical site staff, and patients. We further emphasize the need for individual biosafety considerations for distinct types of OV immunotherapies on a case-by-case basis and urge that regulations and practices must reflect the most up-to-date scientific findings regarding their handling.

## Conclusion

In summary, HSV-1–based OV immunotherapies have been genetically modified for tumor selectivity and safety, rendering them vastly different from the wild-type virus. Despite this attenuation, these agents are often assigned the same BSL precautions as the wild-type virus due to concerns of off-target effects and contact transmission. However, as more clinical data become available, it is increasingly evident that some of these OV immunotherapies pose little risk to healthcare workers and close contacts of patients receiving treatment. With the increasing clinical use of OV immunotherapies, ensuring safe yet practical implementation of preparation and administration procedures will be important to ensure that patients have access to these innovative and potentially life-saving medicines. Further individual considerations should be given regarding the origin of the OV immunotherapy (human vs. non-human pathogen), the availability of antiviral treatments, the possibility for viral recombination, and the risk of communal transmission.
